# Barriers to and Facilitators of the Evaluation of Integrated Community-Wide Overweight Intervention Approaches: A Qualitative Case Study in Two Dutch Municipalities

**DOI:** 10.3390/ijerph13040390

**Published:** 2016-03-31

**Authors:** Tessa M. van Koperen, Anja de Kruif, Lisa van Antwerpen, Anna-Marie Hendriks, Jacob C. Seidell, Albertine J. Schuit, Carry M. Renders

**Affiliations:** 1 Department of Health Sciences, Faculty of Earth and Life Sciences, Section Prevention and Public Health and EMGO+ Institute for Health and Care Research, VU University Amsterdam, De Boelelaan 1085, Amsterdam 1081 HV, The Netherlands; carry.renders@vu.nl; 2 Department of Health Sciences, Faculty of Earth and Life Sciences, Section Methods and Applied Biostatistics, Qualitative Research, VU University Amsterdam, De Boelelaan 1085, Amsterdam 1081 HV, The Netherlands; anja.de.kruif@vu.nl; 3 Youth on Health Weight, Causariestraat 5, Den Haag 2511 VB, The Netherlands; lisa.vanantwerpen@jogg.nl; 4 Department of Health Promotion, Faculty of Health, Medicine and Life Sciences, Maastricht, Maastricht University, Universiteitssingel 60, Maastricht 6229 ER, The Netherlands; anna-marie.hendriks@maastrichtuniversity.nl; 5 Department of Health Sciences, Faculty of Earth and Life Sciences and the EMGO+ Institute for Health and Care Research, VU University Amsterdam, De Boelelaan 1085, Amsterdam 1081 HV, The Netherlands; j.c.seidell@vu.nl; 6 National Institute for Public Health and the Environment, PO Box 1, Bilthoven 3720 BA, The Netherlands; jantine.schuit@rivm.nl

**Keywords:** evaluation, barriers, facilitators, perceptions, case study, community, overweight

## Abstract

To prevent overweight and obesity the implementation of an integrated community-wide intervention approach (ICIA) is often advocated. Evaluation can enhance implementation of such an approach and demonstrate the extent of effectiveness. To be able to support professionals in the evaluation of ICIAs we studied barriers to and facilitators of ICIA evaluation. In this study ten professionals of two Dutch municipalities involved in the evaluation of an ICIA participated. We conducted semi-structured interviews (*n* = 12), observed programme meetings (*n* = 4) and carried out document analysis. Data were analyzed using a thematic content approach. We learned that evaluation is hampered when it is perceived as unfeasible due to limited time and budget, a lack of evaluation knowledge or a negative evaluation attitude. Other barriers are a poor understanding of the evaluation process and its added value to optimizing the programme. Sufficient communication between involved professionals on evaluation can facilitate evaluation, as does support for evaluation of ICIAs together with stakeholders at a strategic and tactical level. To stimulate the evaluation of ICIAs, we recommend supporting professionals in securing evaluation resources, providing tailored training and tools to enhance evaluation competences and stimulating strategic communication on evaluation.

## 1. Introduction

Childhood obesity is a global public health problem [1,2]. In 2013, 23.8% of the boys and 22.6% of the girls in developed countries were affected by overweight or obesity and in developing countries these percentages were 12.9% and 13.4% respectively [3]. Although the increase in the prevalence of childhood obesity appears to be levelling off in some countries [4,5], in most countries the increase continues [6]. Obesity is associated with an increased risk of serious complications such as type 2 diabetes and cardiovascular diseases but also with psychosocial problems and a reduced quality of life [7,8]. Moreover, childhood obesity often persists into adulthood [9,10].

Governments and communities worldwide try to prevent obesity, starting in childhood or even before birth [11]. Traditionally, such prevention approaches focused on individual health determinants such as the motivation of children to be physically active on a daily basis, or the lack of knowledge about healthy eating [12]. Unfortunately, these individually targeted prevention efforts showed disappointing outcomes [13,14]. This seemed related to the fact that these individual determinants of overweight and obesity were targeted, while the social and physical environment in which children grow up remained the same and therefore did not stimulate a healthier lifestyle [15]. Experts are now advocating the implementation of an integrated community-wide intervention approach (ICIA), in which both personal as well as environmental determinants are targeted. Such an approach should include a mix of interventions working in conjunction with each other in the settings where children live, learn, and play (early care and education, home, school, community, health care), should be directed towards multiple target groups in the community, and should share a long-term health related goal. Subsequently, it should be implemented by sectors within and outside the health domain [16,17,18,19,20].

**Figure 1:**
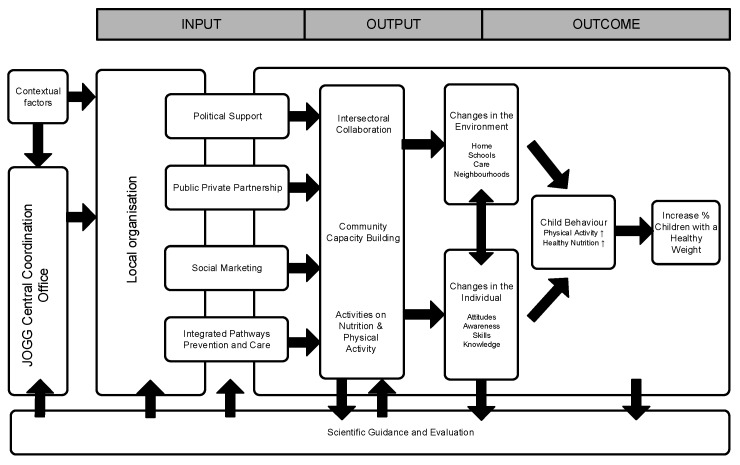
JOGG model.

A successful example of an ICIA is the EPODE approach (EPODE stands for “Ensemble Prévenons l’Obésité Des Enfants” or “Together Let’s Prevent Childhood Obesity”), which was implemented in 15 countries including the Netherlands, where it was named “Youth on Healthy Weight” (In Dutch: “Jongeren op Gezond Gewicht” or the JOGG-approach) [21,22,23,24]. The strength of this originally French approach lies in five critical elements: (1) broad political commitment (*i.e.*, from health and non-health sectors) and integrated (public health) policies; (2) commitment of and collaboration between public and private stakeholders; (3) use of social marketing techniques in intervention design; (4) scientific evaluation and (5) integrated pathways of prevention and care. Between 2009 and 2014, 75 municipalities adopted this approach in the Netherlands, and this number continues to rise. To assist local professionals involved in the JOGG-approach with designing, implementing, evaluating and the advocacy of the approach towards stakeholders a logic model was developed, based upon the EPODE logic model [25] (see Figure 1). Other instruments offered by the National Coordination Office of the JOGG-approach (JOGG-office) to support professionals in the evaluation of their ICIA were (1) an evaluation training for programme managers and epidemiologists; (2) an evaluation manual including multiple instruments and tools to support the evaluation process and data collection and (3) the possibility of involving an evaluation expert.

Unfortunately, early experiences with the JOGG-approach show that the critical element “scientific evaluation” is often neglected within the JOGG-approaches in municipalities, despite the fact that evaluation is recognized as being important in continuously improving the ICIA and in achieving a sustainable and more effective programme [26,27]. Evaluation in the context of an ICIA is broader than simply a systematic assessment of the worth or merit of something. It is “the systematic acquisition and assessment of information to provide useful feedback about some object” [28]. Hence, it relates to the total process of formative and summative evaluation, which already commences when an intervention is initiated and can be used to improve the intervention during its course. In ICIAs an evaluation process takes place within a political and organizational context, it requires group skills and capacities, management ability, political leverage and sensitivity to the needs of multiple stakeholders. Evaluation is therefore different from research which is grounded in experimental methods and whose goal it is to create new scientific knowledge. A classical way to describe this difference between evaluation and research is given by Patton: “research seeks to prove, evaluation seeks to improve…” [29].

Previous studies suggest that a possible barrier to the evaluation of complex community-wide approaches is the difficulty in selecting an appropriate design and methodology for evaluation [30,31,32,33]. Other studies indicate that the evaluation of these initiatives is hampered because evaluation is perceived differently by the various stakeholders involved [34,35] and performing such an evaluation needs to compete with other priorities for scarce available resources (e.g., time, budget) [25,36,37]. A limited number of studies have explored barriers to and facilitators of evaluation. However, to the best of the authors’ knowledge no studies have involved professionals engaged in an ongoing ICIA [38,39,40].

In order to improve support to those implementing ICIAs across cases, in-depth understanding about barriers to and facilitators of evaluation of ICIAs is needed. This understanding can then improve planning and execution of evaluation of these comprehensive approaches and hence may inform public policy and practice on optimization of the use of ICIAs. Therefore, we explored the perceptions of professionals involved in an ICIA with regards to barriers to and facilitators of the evaluation of an ongoing ICIA.

## 2. Materials and Methods

### 2.1. Design

A qualitative case study design was used to collect data in two Dutch municipalities (our cases) that implemented the JOGG-approach. Since our goal was to explore a wide range of barriers and facilitators we chose to combine semi-structured interviews with observations and a document analysis among a heterogeneous sample of municipalities. This design supports the explorative nature of this study [41]. The proposal was submitted to the medical ethical committee of the VU medical centre, which judged that a waiver of medical ethical approval was applicable for this explorative study on perceptions of professionals about evaluation of ICIAs.

### 2.2. Sampling

To recruit our two cases, we emailed a study participation request to six eligible municipalities that had implemented the JOGG-approach in February 2014. Inclusion criteria were: one large and one small municipality; with preferably different initiators of the JOGG-approach, which began implementation at least two years ago; affiliation with different Regional Public Health Services (RPHS); and no participation in another external research programme. Based on these criteria, we selected two municipalities (hereafter called Case A and Case B).

From the two cases, we used emergent sampling to select a heterogeneous group of respondents based on position (e.g., programme managers, RPHS epidemiologists, representatives, policy advisors) and degree of involvement within the JOGG-approach (e.g., involved in daily operations, operating at strategic or at tactical level). Emergent sampling implies that our study population emerged and unfolded while the study progressed, rather than being constructed prior to the study. We started with purposive sampling for initial response and because it requires the knowledge of insiders to locate respondents for the study we used snowball sampling to identify other respondents. Snowball sampling means that we asked the first purposively sampled respondents for additional relevant contacts who would be able to provide different or confirming perspectives [42,43]. All respondents employed by the municipality were from the Department of Health.

In Case A, no prior ICIAs had been undertaken to prevent children getting affected by overweight and obesity. A multinational corporation that headquartered in the municipality initiated the JOGG-approach by convincing the municipality to implement the JOGG-approach. Case A was a medium sized urban municipality in the Netherlands of approximately 25,000 inhabitants. The JOGG-approach was implemented in the whole municipality. In Case B the municipality initiated the implementation of the JOGG-approach as a follow-up to an existing integrated approach to the prevention of lifestyle problems, including overweight. Case B was a somewhat larger urban municipality in the Netherlands with approximately 55,500 inhabitants. In this municipality, a community approach was chosen in which two neighbourhoods were involved of approximately 7400 people in total. In contrast to Case A, no other municipality that had implemented the JOGG-approach was affiliated with the RPHS in Case B.

### 2.3. Data Collection

#### 2.3.1. Interviews

Interviews were conducted with five respondents in each case (LvA). Respondents had positions within the municipality and within the RPHS at strategic (or administrative), tactical (or managerial), and operational (or executive) level. The duration of the interviews ranged from 43 min to 67 min. As presented in Table 1, the total amount of hours available for the JOGG-approach was higher in Case B than Case A, respectively 33.20 h and 10.45 h a week. 

Furthermore, the programme manager and policy advisor for Case B were on average ten years older than the ones for Case A. Respondent four of Case A is indicated as “Health Promotion Professional” and respondent four of Case B is indicated as “RPHS-employee” (see Table 1). Prior to the interview, all respondents signed an informed consent. Interviews were conducted with a topic list. This topic list was based on literature and the model of Preskill and Boyle [44]. Additionally, three researchers (LvA; AdK; MvK) developed the topic list collaboratively and carried out a pilot test. The topic list evolved over time as the interview process continued. Topics focused on the role of a respondent in evaluation, evaluation competence (knowledge, skills, and attitude), perception of the progress of evaluation, resources for evaluation, organizational structure of the municipality, key persons in evaluation, and perceived collaboration.

Firstly, programme managers and policy advisors of Case A and Case B were interviewed. In Case A, LvA also attended an introductory meeting at tactical level with a new account manager employed by the JOGG-office. During this meeting the account manager asked questions of the program manager and policy advisor to outline the current situation of the JOGG-approach in the municipality. The responses from the interviews from Case A were supplemented with shared information. Insight was gained into organisational structure and the status of the JOGG-approach in the municipalities. Based on the results of the first four interviews, complementary interviews were performed with other stakeholders at Case A and Case B. As the interview process progressed, the programme managers were interviewed again to further elaborate on the evaluation of activities and responsibility for evaluation. After each interview, LvA wrote a report to record the first impressions and a brief description of the respondent and interview location, the course of the interview, summary, and key words. The recordings were transcribed verbatim. Respondents received a summary of the interview in order to check the credibility of the findings (member check) [45].

#### 2.3.2. Observations

Observations were conducted during meetings at strategic programme level (e.g., steering committee) and operational programme level (e.g., working groups) of both the JOGG-approaches. In Case A, the researcher (LvA) conducted an observation at operational project level. In Case B two observations were conducted at the strategic project level and one at the operational project level. Notes regarding the setting, actions, mutual power relationships, mode of interaction, content, decision-making, and non-verbal cues of all stakeholders were made, and the extent to which stakeholders participated during the meetings. Afterwards, LvA continued informal discussions with stakeholders where possible (e.g., during a car ride). In this way, she was able to strengthen the relationships she had established in earlier interviews and was able to ask questions in a more informal way. After each day of observation, LvA made comprehensive field notes of her observations and informal discussions. These detailed notes generated insight and better understanding of the data collected during the interviews.

#### 2.3.3. Document Analysis

All interviewed respondents were asked to share documents outlining the project structure and current situation of the (evaluation of the) JOGG-approach and documents containing information about the embedding of the JOGG-approach in politics. A variety of documents were reviewed in each case: programme plans, minutes of meetings (at strategic and operational level), overviews of resolutions of the Municipal Board, Memorandums of Public Health, newsletters, and organization charts. The information extracted from the documentation was used in two ways: (1) as input for interviews or to highlight situations that needed to be observed; and (2) to cross-validate data gathered during the interviews and observations [46].

### 2.4. Data Analysis

Concepts from the multi-disciplinary model of evaluation capacity building (ECB) of Preskill and Boyle [44] were used in the development of the coding scheme. Although our research does not focus on ECB itself, exploring the barriers to and facilitators of performing evaluation may increase insight into what is needed to build evaluation capacity among professionals in ICIAs. This conceptual framework suggests multiple individual and organizational resources that should be in place in order to build local evaluation capacity and establish a sustainable evaluation practice [44]. When these resources are not in place a sustainable evaluation practice is not feasible.

The interview transcripts and observations were analysed by two researchers (LvA, AdK) using thematic content analysis [47]. The transcripts were read through several times. The texts were divided into fragments, and codes (labels) were assigned to these fragments (open coding). These codes were all organized into a mind map. The preliminary conclusions were thoroughly discussed with MvK. The last phase of the analysis was selective coding: the essence of what each theme was about was identified, searched for relations through constant comparison across cases and analysed variation within and between cases. Finally, the different themes were fitted into the broader picture of the entire data. During the entire analytical process, memos were used to record thoughts about the results and to distinguish between the researchers’ interpretations and respondents’ own views. To ensure validity and reliability thoughts and analysis were discussed within the research team.

## 3. Results

### 3.1. Case Description

Both cases had adopted the JOGG-approach in the same year, 2012, but with different initiators and point of departure. In Case A, a multinational corporation that headquartered in the municipality brought the JOGG-approach to the attention of the alderman, who in turn supported the implementation in order to maintain a good relationship with this private company. No preparations had been undertaken prior to the JOGG-approach: the municipality started from scratch, as reflected by the programme manager’s perception of her function:

“*Sometimes (I have) the feeling of: ‘where to start?’ and (it feels like) droplets in the ocean*” (A1).

In Case B, the initiator of the JOGG-approach was the municipality. A programme similar to the JOGG-approach, called programme X in this study, had already been implemented. Programme X was an integrated approach to the prevention of lifestyle problems, including overweight, and was accompanied by financial and substantive support. Therefore, a certain knowledge and experience base was already established. According to the programme manager the JOGG-approach was used to amplify the previous programme.

In both cases a steering group and multiple working groups were established. At strategic level both JOGG-approaches were coordinated by an alderman and the department head of the health sector of the municipality. In both cases a “steering group” functioned more on the operational level than on the strategic level. A respondent from Case A described the steering group as having a lack of:

“*… real vision, policy, and making decisions (at strategic level)”* (A1).

More people were part of the project structure in Case B than in Case A. For example in Case A eight people were part of the working group “Interventions” and in Case B there were twelve. The steering group of Case A had seven members and in Case B eleven. Case A focused its programme structure around “Interventions” and “Communication”. And Case B focussed its programme structure around “Interventions”, “Care and prevention”, and “Evaluation”.

The interviewed professionals in Case A were younger and had not been in the employment of their organisations for as long as those in Case B and were less experienced with evaluating public health initiatives. Moreover, working time (hours) for the JOGG-approach was higher in Case B and in this case more professionals from in and outside the local government and at different levels (strategic, tactical and operational) were involved*.*

In both cases, no evaluation team was established even though this was recommended in the JOGG evaluation manual and in the first (of four) evaluation training session of the JOGG-office. The programme manager and the epidemiologist in both cases attended this first training session. The programme manager of Case B explained they did not establish a separate evaluation team, but made evaluation part of existing working groups. It appears this was a non-formalised agreement since all respondents of Case B were involved in evaluation and had their own strategic and operational tasks and roles. The programme manager of Case A said they doubted the need for such a team, said a clear mandate for such a team had not been given by the steering group, and there were no resources for evaluation.

### 3.2. Evaluation Barriers and Facilitators

In the upcoming paragraphs we describe our merged results from Case A and Case B. Perceived barriers to and facilitators of evaluation in each case are presented using direct quotations from interviews (indicated by italic letters and enclosed in quotation marks), field notes, observations, and documents (enclosed in quotation marks). A total of six themes derived as key-issues after data-collection: motivating factors to evaluate, perceived feasibility of evaluation, knowledge and attitudes of evaluation, communication and involvement with evaluation, evaluation resources, and support from decision-makers. An overview of the results can be found in Table 2.

#### 3.2.1. Motivating Factors to Evaluate

Analysis of data showed that a trigger or motivating reason could give rise to an evaluation (Case B). On the other hand, the lack of it could impede evaluation (Case A). All interviewees asked said a specific person was needed to motivate evaluation. In particular the programme manager was seen as having an ideal position to mobilise and motivate professionals:

*“The programme manager is an enthusiastic person who can transfer her energy properly. So that works very well”* (B4).

The programme manager mentioned (Case B) also seemed to understand her role to trigger evaluation processes since she asked for evaluation-related activities*:*

*“My role (as programme manager) is to question (..) them (the stakeholders) about the benefit of their activities. So in that way we try to collect data (about evaluation)”* (B1).

In Case B, the alderman also provided such a trigger by asking for an effect evaluation. Moreover, the programme manager and policy advisor in Case B said an external public funder of programme X provided an additional trigger by requesting a process evaluation in a final report for accountability reasons. Also the evaluation expert who was made available to municipalities by the JOGG-office was a catalyst in the design and execution of evaluation and helped others to understand the evaluation process.

*“(…) it was good to have the evaluation expert from the JOGG-office visit us and discuss the evaluation possibilities and effects to be shown at local level (...), this works better than the training sessions alone, which are more general*” (B1).

However, this programme manager would have liked more directives on evaluation from the JOGG-office. In contrast, interviewees in Case A said no-one triggered evaluation. To stimulate evaluation, the policy advisor and health promotion professional from Case A therefore suggested that an epidemiologist from the RPHS, the JOGG-office, a programme manager or the alderman should ask for evaluation. The evaluation training sessions were not perceived as stimulating the programme manager of Case A to commence evaluation planning:

*“(…) the training sessions were too formal, extensive, detail oriented and exaggerated”* (A1).

The given information was too overwhelming for her and did not increase her motivation to study the process of evaluation let alone stimulate her to design and perform the evaluation of the JOGG-approach.

#### 3.2.2. Perceived Feasibility of Evaluation

Interviewees in both cases perceived evaluation of the JOGG-approach as unfeasible. They assumed that a sound and proper evaluation could not be performed with their limited budget, time and capabilities. For example, the programme manager of Case A and policy advisor of Case B said:

“*There is so much to it (evaluation) if you want to do it well. A lot of time, money (..), and expertise*” (A1).

*“If you want to do it (evaluation) properly, you have to make a big investment”* (B2).

Most interviewees added that the evaluation as presented in the evaluation training and in the evaluation manual of the JOGG-office represented an “ideal” evaluation which was not encountered in practice or was even incompatible with practice. Some interviewees struggled with the translation of the evaluation manual for use in their own situation. Both the epidemiologist and the RPHS-employee of Case B explained that evaluation of the JOGG-approach was not systematic, but “emerged” during implementation:

*“I do notice that practice is more stubborn (than the ideal situation), so then you’ll have to look further and search for your own pathway”* (B3).

*“You want to write things down as specifically as possible in such an (evaluation) plan. However, JOGG is also characterized by (..) a bottom-up approach and setting goals together. And sometimes I perceive that as quite a tension”* (B4).

#### 3.2.3. Knowledge of and Attitudes to Evaluation

In both cases the programme managers indicated they lacked sufficient knowledge of evaluation. The epidemiologists were believed to be sufficiently equipped to design and perform evaluation. However, the policy advisor in Case A said that some epidemiologists who were believed to be capable of designing and conducting an evaluation were in reality not able to do so. This might be explained by the fact that most interviewees, including the epidemiologists, did not understand the difference between evaluation and research, they used the terms interchangeably, hence making evaluation bigger and more difficult than necessary. For instance, the programme manager and policy advisor of Case A frequently used the terms evaluation and research interchangeably and referred to the working group “Research” when the interviewer asked about evaluation. Asking about her competences in evaluation, the programme manager of Case A replied:

*“I did some research during my study but not like a youth monitor or something. I would not know what questions to ask or when such a research is representative or reliable (…)”* (A1).

Moreover, interviewees in Case A did not perceive a process evaluation in the form of an oral communication of an activity to discuss the progress and possible areas of improvement, as part of the programme evaluation, because it was not formal enough. In Case B, the programme manager, epidemiologist, and RPHS-employee knew that process evaluation was important to optimize the approach, but their attitudes to the process evaluation were less positive:

“*it has no scientific value*” (B2) and “*it is not something official*” (B4).

The epidemiologist felt that she and the other professionals were not fully equipped to conduct a process evaluation. Additionally, the programme manager and RPHS-employee of Case B showed they were aware of differences in evaluation between different initiatives, they said evaluation could either be extensive or superficial depending on needs and funding.

In contrast to the different purposes of evaluation and research as discussed in the introduction to this paper the programme manager, policy advisor, and health promotion professional of Case A all mentioned that evaluation findings could be used to “prove” something. These perceptions on evaluation can limit the use of evaluation for programme optimization and diminish the value of evaluation at strategic and tactical levels.

Regarding respondents’ attitudes towards evaluation, some respondents questioned whether the effects observed could be allocated to the JOGG-approach. The policy advisor and epidemiologist of Case B, for example, explained it would be difficult to produce “hard” data about the relationship between the JOGG-approach and effects. Furthermore, several respondents cited that evaluation required a lot of investment of resources. One respondent commented:

“*And it is the question whether it (the evaluation) is worth the investment*” (B2).

Evaluation in general was considered important by the majority of the respondents:

“*When you evaluate, you know what works and what does not (..). And you can keep partners on board, show results, (create) a positive atmosphere, and attract other parties*” (A1).

“*It is important to see whether what you do achieves effects*” (B2).

#### 3.2.4. Evaluation Resources

Data revealed that lack of time and allocated financial resources prohibits the evaluation of the JOGG-approach (Case A). On multiple occasions the programme manager of Case A stated that limited financial resources were available to administer questionnaires, to hire personnel for data collection and analysis, and to set up a proper evaluation. In this municipality a budget for evaluation of the JOGG-approach was not generated. Since the RPHS monitors health and behaviour of children in the municipality it was decided to use this data to show results, despite the limitations of this data. In Case B, on the other hand, allocated time and financial resources were believed to have stimulated the evaluation process:

“*Within the entire process we have allocated time and money in order to conduct a proper evaluation*” (B2).

Interviewees in Case A reported that the annual fee of the JOGG-approach was paid by the multinational headquartered in the municipality, while in Case B, the annual fee was funded by the subsidy for programme X and reserved resources from local health policy. Concerning time (personnel resources), hours were allocated for an epidemiologist in Case B, whereas no additional hours for evaluation were made available in Case A. The programme manager of Case A believed evaluation could be stimulated by the involvement of someone with substantive knowledge of research who could lead the workgroup Research. Regarding material resources, the JOGG-office provided various materials to support evaluation: an evaluation manual, the JOGG-model, process guidelines, an activity monitor (a digital monitor to register all activities), and a parental questionnaire on children’s health and behaviour. Although the parental questionnaire was used in both cases, the remaining materials were not (frequently) used by the respondents. The programme managers and epidemiologists described some issues that discouraged them from using the activity monitor:

*“The development process of the activity monitor by the JOGG-office took a long time and it still is not finalized”* (B3),

*“It is not really a useful tool”* (B1;A1).

*“The activity-monitor has limitations in space to insert all retrieved data*” (B3).

and

*“*(…) o*ther JOGG-municipalities do not use it”* (A3).

*“It is not easy to use and needs an update in lay-out”* (A3).

#### 3.2.5. Communication and Involvement with Evaluation

**Figure 2:**
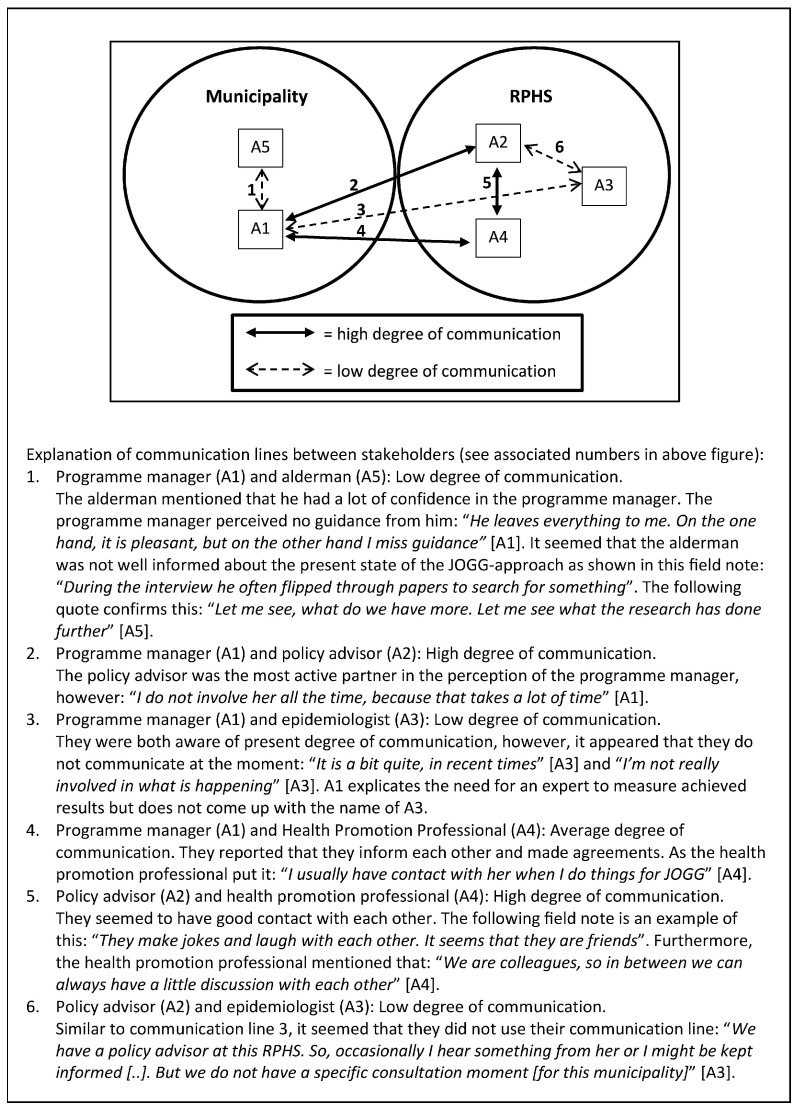
Communication flow of Case A.

**Figure 3:**
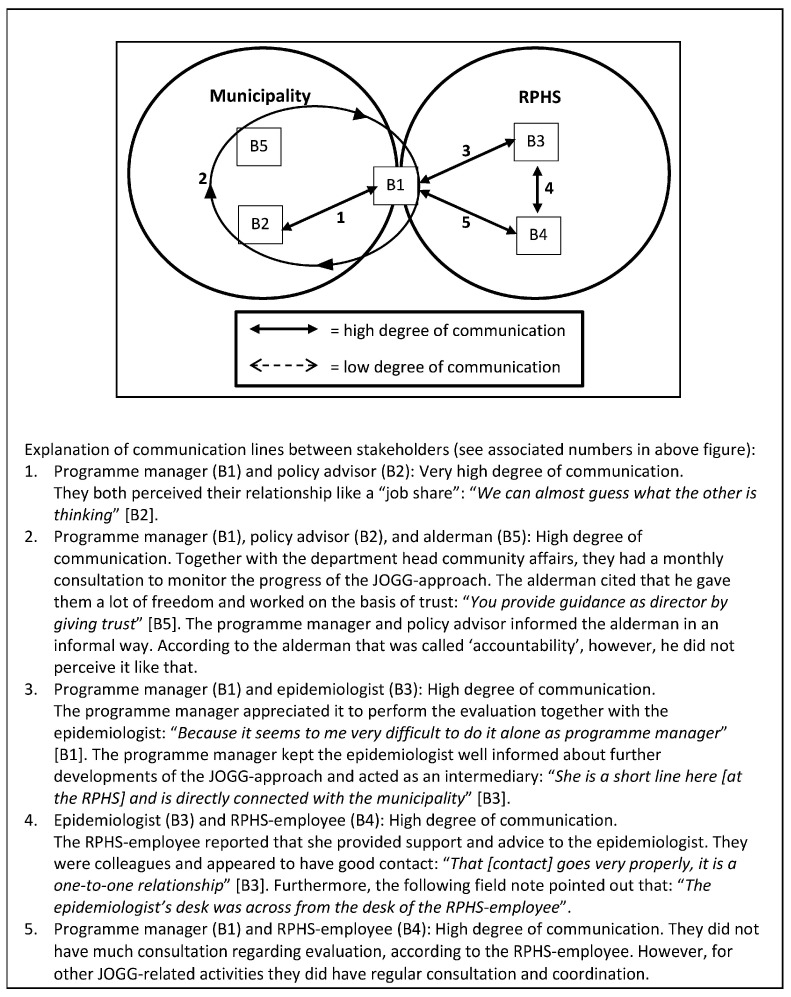
Communication flow of Case B.

Communication was considered key in the evaluation of an ICIA. When there is no communication on evaluation professionals seem less committed to the evaluation process. Communication between the programme manager and epidemiologist was facilitative for evaluation, since it enhanced the involvement of both in the evaluation (see Figure 2 and Figure 3):

*“If you are really involved then it is easier to see where evaluation can do something or add something”* (A3).

In Case A, the programme manager and epidemiologist admitted that they communicated very little, whereas a high degree of communication took place in Case B. In the programme plan of Case B, it was stated that the programme manager had to be the one that connected the municipality and the RPHS. She should communicate with all members of the programme structure, at strategic, tactical and operational levels, inside and outside the municipality structure:

“The programme manager acts as a spider in the web, both inside and outside the municipality” (text fragment drawn from action plan).

#### 3.2.6. Support from Decision Makers

Support from decision makers at multiple levels was important for evaluation since it affected the availability of resources. One epidemiologist explained:

*“One cannot do everything, it is really quite broad. So at a certain point, choices need to be made”* (B3).

Decision makers who were best able to create a support-base for the JOGG-approach were situated at tactical and operational levels inside and outside the municipality. Alderman and city council members were especially important in stimulating the allocation of evaluation resources. In both cases competing priorities amongst politicians and policy makers could reduce attention for the JOGG-approach and subsequently resulted in fewer resources being available for evaluation. Although the approach itself was supported by politicians and other policy makers in Case B, the policy advisor and epidemiologist of Case B cited that evaluation was not always considered important by politicians, although:

*“Sometimes they do think (evaluation) is important and then they say: ‘make money available’. So, that is always a little between the devil and the deep blue sea”* (B2).

To create political support, the programme manager, policy advisor, and alderman of Case B reported that they linked the JOGG-approach to the Social Support Act and drew up a collective policy plan. This was conducive to cooperation with other policy sectors according to the policy advisor.

In Case B the JOGG-approach was mentioned several times in the list of resolutions of the Board of Mayor and Aldermen which shows political support. In addition, the programme manager of Case B appeared to be very pro-active, as illustrated by the following field note:

“*The programme manager went to the financial department on the day it became clear that additional money was needed for a JOGG-approach related activity*”.

In Case A the JOGG-approach was not linked to other formalised initiatives in the municipality and the JOGG-approach itself was only referred to a single time in the resolutions of the city council. Support from politicians for evaluation in Case A was considered low by respondents, the epidemiologist of Case A even believes that municipalities in general are only interested in long-term goals and not in mid-term effects or process evaluations.

The administrative and policy process to generate extra funding for the evaluation for the JOGG-approach would, according to the programme manager, absorb all personnel hours:

*“If you really need money, then you can (write) entire plans and (go) to the Municipal Council. However, then my eight hours a week (for the JOGG-program) are lost due to that”* (A1).

Besides support from policymakers, obtaining support for the evaluation from those who implement the JOGG-approach was considered key to evaluation. However, in both cases obtaining such support was often limited due to different interests, backgrounds, identities, cultures, and origin of implementers. Therefore, having a common goal was seen as facilitative for evaluation in Case B. However, in Case A having a common goal did not emerge from the data. In Case B interviewees actively brought evaluation to the attention of stakeholders, while this did not happen in Case A:

“*We have actually never discussed evaluation (in the working group Interventions)*” (A4).

The RPHS-employee in Case B added that evaluation was not a natural step for stakeholders and not many stakeholders asked for it. To overcome this barrier, the RPHS-employee explained that the importance of evaluation could be made clearer to them. However, the programme manager of Case A did not have the intention to invest in such a process:

“*I’m not going to make a whole action plan to create a support base for research among politicians and private partners. Then it is better to invest my energy in performing those activities and setting up and organizing working group meetings. Because at least something happens then*” (A1).

Besides politicians and implementers, the RPHS was often discussed as important to support evaluation in both cases.

*“I really liked working with the RPHS epidemiologist (…). I think that this is also an advice to other municipalities, ensure that employable hours are made available within the RPHS or for another researcher who can work with you”* (B1).

However, in Case A this imposed a barrier since the budget for the evaluation of the JOGG-approach had not been allocated. According to the policy advisor and epidemiologist it could be possible to increase employable hours of RPHS personnel for the JOGG-approach when it was considered important by the RPHS. Unfortunately, they noted that since the RPHS is a non-profit organisation, support for evaluation would depend on allocation of municipal resources.

*“Employable hours just cost money, (…) so the RPHS cannot just donate employable hours to the municipality*” (A1).

To overcome this evaluation barrier, the epidemiologist suggested creating a clear policy or vision statement at the RPHS about how important support of the JOGG-approach is and how many hours they would like to invest.

## 4. Discussion

In this study we explored the barriers to and facilitators of the evaluation of ICIAs. We believe that these barriers and facilitators can be encapsulated in three themes: the need for an evaluation motive, evaluation feasibility and commitment to evaluation from stakeholders (see Table 2 in result section).

Firstly, the *absence of a clear evaluation motive* can hamper evaluation. Evaluation requires a personal or environmental factor that triggers evaluation. For example, when professionals believe an evaluation has no benefits and people around them (e.g., aldermen) do not provide incentives to evaluate, there is no urge to spend scarce resources on evaluation. Other studies refer to this as the “demand side” of evaluation [48] or “readiness for evaluation” [49]. This readiness for evaluation could also be a function of involved professionals with more experience.

Secondly, *perceived feasibility* may hamper evaluation. Two main factors determined feasibility: evaluation skills and opportunities. ICIA evaluation requires continuous realigning of “theory” and “practice” of evaluation since an ICIA emerges during implementation, requires a focus on the process (besides the effect), and is often “bottom-up”. Professionals might be capable of conducting a “top-down” evaluation which is planned in a more linear way. However, for ICIA evaluation, professionals need to empower the community (*i.e.*, stakeholders) to evaluate [50,51] and make adaptations during the evaluation process. Therefore, skills are required that enable the professional to design, plan and conduct both an effect *and* process evaluation and work within an emerging environment. Besides such a broad set of evaluation skills, time and financial resources need to be sufficient to make the evaluation feasible. Lack of time and financial resources are a commonly found evaluation barrier and particularly pervasive when it concerns the evaluation of ICIAs (compared to a less comprehensive evaluation) [25,37,40,52,53]. Professionals therefore need to act as “jugglers”: to prioritize evaluation in a context with multiple competing priorities and limited evaluation capacity [48]. In order to take upon the roles involved professionals need to be highly experienced in Public Health.

Thirdly, the (lack of) *commitment to evaluation from stakeholders* can hamper evaluation. Such commitment is necessary to enhance the evaluation capacity and is primarily created through communication and trust: *“many of the problems encountered by evaluators, much of the resistance to evaluation, and many failures of use occur because of misunderstandings and communication problems”* [42,44]. Therefore, communication that is specifically focused on building trust and relationships [38] between the programme manager and a person with expertise in evaluation is required. Professionals also need to communicate effectively with the community and decision makers to keep them involved in the evaluation process and to understand the importance of evaluation. Such communications skills are even more important since obtaining evaluation support from decision makers at strategic (politics), tactical (managerial) and at operational level (in municipality and the RPHS) was found to be extremely difficult, but is also an essential element for ICIAs (*i.e.*, “political commitment” and “public private partnership”) [23].

Furthermore, in the Netherlands the Regional Public Health Services have long since provided municipalities with epidemiological data on public health. This makes the RPHSs and health sectors of municipalities very outcome driven with public health initiatives with pre and post-measurements on sickness and health. Within the Dutch Public Health there is no tradition of seeing evaluation as a continuous process that should be aligned with the design and implementation of specific programmes, let alone in comprehensive community-wide interventions approach. Therefore it seems logical that epidemiologists, that are used to show effects (hard, quantitative data) of a public health intervention, indicate they have a lack of trust in their capabilities to use evaluation in emerging practice. However, we do feel that this study showed multiple factors that hamper the very necessary evaluation for ICIAs and therefore we advise strengthening epidemiologists and the managers of these programmes with knowledge and skills in programme evaluation and evaluation support from managerial level and funders to enhance the successes of these comprehensive programmes.

One of the strengths of this study is its design: a case study research is useful in order to gain a rich, detailed understanding of evaluation within a real life context [54]. Although we explored two cases the views of the respondents might not be reflective of the perceptions of stakeholders in other JOGG-municipalities. However, we feel the examination of the topic at hand within a real life context increases the reliability and ecological validity of the results [55]. In addition, the explorative and qualitative nature of this study can provide a deeper insight into evaluation of the integrated approach compared to a quantitative study. Finally, it appeared afterwards that some stakeholders might not have fully understood the difference between evaluation and research. This may put the perceptions of these respondents in a different perspective. At least the present study provides insight into the existence of this lack of understanding.

## 5. Conclusions

Evaluation remains an important component of ICIAs but in practice it is often neglected. Professionals such as programme managers and epidemiologists appear to have no motive to evaluate, and lack knowledge, skills and resources to evaluate an ICIA resulting in poor evaluation feasibility and lack of commitment to evaluation from stakeholders. As such, evaluation capacity is insufficient and needs to be enhanced. This could be achieved by providing an incentive for evaluation, supporting programme managers to generate and allocate sufficient time and financial resources to programme evaluation, communicating to them the difference between evaluation and research, and through teaching or supporting programme managers and epidemiologists to invest in commitment of stakeholders at multiple levels inside and outside local government.

The incentive for evaluation of an ICIA can be provided by programme funders (*i.e.*, demanding 10%–15% evaluation budget in programme plans) and by central or national programme coordinators (*i.e.*, compulsory training in program evaluation for programme management; the provision of an evaluation coach to stimulate evaluation planning, communication on evaluation and assist in the appointment of a responsible evaluation manager). The incentive can also be part of organizational policy with regard to evaluation within local government and RPHS, or through support from the tactical and strategic level for programme evaluation within these organisations. We also urge central coordinated programmes such as JOGG that are known to persuade and support municipal governments to develop and disseminate local ICIAs to discuss the importance of programme evaluation for the success of the ICIA with municipalities to increase evaluation budget and motives for evaluation of the ICIA beforehand. By investing in commitment from stakeholders and incentives for evaluation, the quantity and quality of evaluation of these comprehensive approaches may improve the design and the implementation of the ICIA, the sustainability of the approach and strengthen the evidence base of effective prevention strategies for childhood obesity.

Further insight is needed to establish a sustainable evaluation practice within organisations involved in designing and implementing ICIAs, and to understand which interventions and policies are here for needed. Future studies should examine different strategies to increase engagement of programme managers, epidemiologists and stakeholders at tactical and strategic level in the programme evaluation of an ICIA.

The ICIA we studied in two municipalities in The Netherlands is based upon the EPODE approach. Although EPODE advocates a community-wide approach to prevent children getting affected by overweight and obesity it is not a one-size-fits-all approach, it still needs to be adapted to the local situation. A programme evaluation can support this adaptation. Due to the specific evaluation culture in the Netherlands we cannot automatically apply the results of this study to other EPODE approaches. We do think that since the EPODE approach is expanding internationally it is important for current and future EPODE approaches but also for other ICIAs, to pay sufficient attention to programme evaluation and its advantages for programme optimization, to evaluation capacity building for programme management and to evaluation funding and evaluation support at tactical and strategic level.

## Figures and Tables

**Table 1:** Individual characteristics of the respondents (*n* = 10).

Respondent *	Function	Gender	Age (Years)	Level of Education	Organization	Years of Service	Working Time for JOGG (Hours) **
A1	Programme manager	F	23	BS	Municipality	2	8.00 weekly
A2	Policy advisor	F	28	MS	RPHS	2	1.25 weekly
A3	Epidemiologist	F	43	PhD	RPHS	4	0.24 weekly
A4	Health promotion professional	F	27	MS	RPHS	4	0.96 weekly
A5	Alderman	M	62	-	Municipality	4	-
B1	Programme manager	F	48	BS	Municipality & RPHS	2 & 13	20.00 weekly
B2	Policy advisor	F	55	MS	Municipality	14	8.00 weekly
B3	Epidemiologist	F	38	MS	RPHS	12	4.00 weekly
B4	Epidemiologist & Policy advisor (RPHS-employee)	F	37	MS	RPHS	11	1.20 weekly
B5	Alderman	M	55	-	Municipality	4	-

* = Letter A represents Case A, letter B represents Case B; ** = Calculated on the basis of 208 workable days (40-hour working week). Abbreviations used: BS = Bachelor of Science, MS = Master of Science, PhD = Doctor of Philosophy, RPHS = Regional Public Health Service.

**Table 2:** Barriers to and facilitators of the evaluation of the JOGG-approach.

Themes	Subthemes	Barriers (−)/Facilitators (+)	Examples
*The need for an evaluation motive*			
Motivating factors to evaluate	Person to motivate evaluation	+	An evaluation expert who provides expertise and support to start the evaluation process
		−	Lack of a person who stimulates performance of evaluation
	Demand for evaluation	+	External funder or alderman to ask for evaluation results
		−	The programme manager does not give a command to start the evaluation process
*Evaluation feasibility*			
Perceived feasibility of evaluation	Assumptions on feasibility of evaluation	+	Existence of a realistic perception of evaluation
		−	Negative perceptions on feasibility of evaluation as presented in theory and evaluation models
	Capabilities of those involved	+	Trust in interpretation of tasks
		−	Lack of trust in capabilities of those that should be involved in the evaluation: programme manager and epidemiologist.
Knowledge and attitudes on evaluation	Positive attitude towards evaluation	+	Evaluation is regarded as important
		−	Doubt about possibilities to show effects of ICIA
	Knowledge on evaluation	+	Availability of a person with sufficient knowledge on what the (process of) programme evaluation implies and how to conduct such an evaluation
		−	Lack of evaluation knowledge (*i.e.*, oral informal process evaluation has no scientific value)
	Perception of own capabilities	+	High self-efficacy to conduct evaluation
		−	Negative perception of one’s own capabilities to evaluate
*Evaluation feasibility*			
Evaluation resources	Financial resources	+	Allocated financial resources for evaluation process
		−	Limited resources to hire personnel, resulting in limited time
	Time	+	Allocated hours for involvement of epidemiologist or evaluation expert
		−	Lack of personnel for data collection; Lack of time for evaluation education
	Availability of evaluation instruments	+	Availability of generic suitable evaluation instruments (*i.e.*, questionnaires, logic model)
		−	Non-functioning or incomplete evaluation instruments
*Commitment of evaluation stakeholders*			
Communication and involvement with evaluation	Communication on evaluation	+	Regular and high degree of communication between programme manager and epidemiologist on evaluation
		−	Low degree of communication on evaluation between programme manager and epidemiologist; Low degree of communication between programme manager and alderman
	Involvement in evaluation	+	Active involvement of stakeholders helps to see the added value of evaluation
		−	Involvement of members of the programme structure at strategic as well as tactical and operational level
Support form decision-makers at multiple levels	Political support (tactical)	+	Evaluation is considered important by alderman and city council
		−	Competing themes that reduce attention and make fewer resources available; Politicians only interested in long-term goals and not in mid-term or process evaluations
	Managerial support (strategic)	+	A pro-active attitude of department management to generate resources and support; a clear policy vision of RPHS supportive to the approach and evaluation
		−	A time consuming policy process to generate extra financial resources
	Support from implementers (operational)	+	Stakeholders have a common goal
		−	Limited interest in evaluation from those who implement the ICIA

## Abbreviations

The following abbreviations are used in this manuscript:

ICIA	Integrated Community-wide Intervention Approach
EPODE	Ensemble Prévenons l’Obésité Des Enfants (French) or “Together Let’s Prevent Childhood Obesity” (English)
JOGG	Jongeren op Gezond Gewicht (Dutch) or Youth on Health Weight (English)
JOGG-office	National Coordination Office of the JOGG-approach
RPHS	Regional Public Health Service
